# The functions of long non-coding RNA (lncRNA)-MALAT-1 in the pathogenesis of renal cell carcinoma

**DOI:** 10.1186/s12882-023-03438-1

**Published:** 2023-12-20

**Authors:** Omid Anbiyaee, Arash Moalemnia, Farhoodeh Ghaedrahmati, Maryam Khombi Shooshtari, Seyed Esmaeil Khoshnam, Bartosz Kempisty, Shahla Ahmadi Halili, Maryam Farzaneh, Olanrewaju B. Morenikeji

**Affiliations:** 1 Cardiovascular Research Center, School of Medicine, Namazi Hospital, Shiraz University of Medical Sciences, Shiraz, Iran; 2 Faculty of Medicine, Dezful University of Medical Sciences, Dezful, Iran; 3 Department of Immunology, School of Medicine, Isfahan University of Medical Sciences, Isfahan, Iran; 4 Chronic Renal Failure Research Center, Ahvaz Jundishapur University of Medical Sciences, Ahvaz, Iran; 5 Persian Gulf Physiology Research Center, Medical Basic Sciences Research Institute, Ahvaz Jundishapur University of Medical Sciences, Ahvaz, Iran; 6 Department of Human Morphology and Embryology Division of Anatomy, Wrocław Medical University, Wrocław, Poland; 7 Department of Veterinary Surgery, Institute of Veterinary Medicine, Nicolaus Copernicus University, Torun, Poland; 8 Physiology Graduate Faculty North, Carolina State University, Raleigh, NC 27695 US; 9 Center of Assisted Reproduction Department of Obstetrics and Gynecology, University Hospital and Masaryk University, Brno, Czech Republic; 10 Department of Internal Medicine, School of Science, Chronic Renal Failure Research Center, Ahvaz Jundishapur University of Medical Science, Ahvaz, Iran; 11 Fertility, Infertility and Perinatology Research Center, Ahvaz Jundishapur University of Medical Sciences, Ahvaz, Iran; 12 Division of Biological and Health Sciences, University of Pittsburgh at Bradford, Bradford, PA USA

**Keywords:** Renal cell carcinoma, lncRNAs, MALAT-1, Biomarker

## Abstract

Renal cell carcinoma (RCC), a prevalent form of renal malignancy, is distinguished by its proclivity for robust tumor proliferation and metastatic dissemination. Long non-coding RNAs (lncRNAs) have emerged as pivotal modulators of gene expression, exerting substantial influence over diverse biological processes, encompassing the intricate landscape of cancer development. Metastasis-associated lung adenocarcinoma transcript 1 (MALAT-1), an exemplar among lncRNAs, has been discovered to assume functional responsibilities within the context of RCC. The conspicuous expression of MALAT-1 in RCC cells has been closely linked to the advancement of tumors and an unfavorable prognosis. Experimental evidence has demonstrated the pronounced ability of MALAT-1 to stimulate RCC cell proliferation, migration, and invasion, thereby underscoring its active participation in facilitating the metastatic cascade. Furthermore, MALAT-1 has been implicated in orchestrating angiogenesis, an indispensable process for tumor expansion and metastatic dissemination, through its regulatory influence on pro-angiogenic factor expression. MALAT-1 has also been linked to the evasion of immune surveillance in RCC, as it can regulate the expression of immune checkpoint molecules and modulate the tumor microenvironment. Hence, the potential utility of MALAT-1 as a diagnostic and prognostic biomarker in RCC emerges, warranting further investigation and validation of its clinical significance. This comprehensive review provides an overview of the diverse functional roles exhibited by MALAT-1 in RCC.

## Introduction

Renal cell carcinoma (RCC) is a type of cancer that originates from the proximal tubule epithelial cells of the renal tubules [1, 2]. According to data from 2018, RCC accounted for approximately 400,000 new cases and 175,000 deaths worldwide [3]. RCC is essentially a metabolic disease characterized by a reprogramming of energetic metabolism [4–6]. In particular, the metabolic flux through glycolysis is partitioned [7–9], and mitochondrial bioenergetics and OxPhox are impaired, as well as lipid metabolism [10–12]. In addition, RCC is one of the most immune-infiltrated tumors [13–15]. Emerging evidence suggests that the activation of specific metabolic pathway have a role in regulating angiogenesis and inflammatory signatures [16, 17]. Features of the tumor microenvironment heavily affect disease biology and may affect responses to systemic therapy [18–21]. The three main histological subtypes of RCC are clear cell RCC (ccRCC), papillary RCC (pRCC), and chromophobe RCC (chRCC). Additionally, there are less common subtypes such as collecting duct RCC, medullary RCC, and RCC associated with hereditary leiomyomatosis and RCC (HLRCC) [22]. Among these subtypes, ccRCC is the most prevalent, representing about 75% of all RCC cases [3]. The risk factors for RCC encompass both genetic and non-genetic factors [23]. Early identification and treatment of renal cancer patients are crucial because conventional chemotherapy and radiation therapies have limited effectiveness against RCC [24]. The primary treatment approach for localized RCC often involves surgical resection of the tumor [23]. Recent studies and progress in the realm of molecular approaches for RCC treatment have showcased various advancements, encompassing immune checkpoint inhibitors, targeted therapies, mTOR inhibitors, combination therapies, genomic profiling, and personalized medicine. In the past few years, the investigation of non-coding RNAs (ncRNAs) as a viable targeted therapy strategy has emerged as a promising and potential avenue for treating RCC. Numerous studies have revealed that long non-coding RNAs (lncRNAs) can function as oncogenes, tumor suppressors, or even exhibit dual roles, depending on the specific context and circumstances [25]. MALAT-1 (Metastasis-Associated Lung Adenocarcinoma Transcript 1) is a lncRNA that has been extensively studied in various cancers, including lung [26], colon [27], breast [28], ovary [29], gastric [30], bladder [31], and hepatic [32]. It is believed that MALAT-1 has been implicated in cancer cell migration, invasion, tumor progression, and metastasis in RCC [33, 34]. MALAT-1 can facilitate epithelial-mesenchymal transition (EMT) in RCC, leading to increased tumor aggressiveness and metastatic potential [34]. High MALAT-1 expression has been associated with advanced tumor stage, metastasis, and reduced overall survival [35]. Despite ongoing research, the functional roles of MALAT-1 in RCC are still under investigation. In the current review, we have compiled and summarized the potential roles of this lncRNA in RCC.

## MALAT-1 biogenesis

MALAT-1, also known by various other names such as Nuclear-enriched Abundant Transcript 2 (NEAT2), HCN, LINC00047, NCRN00047, and PRO2853, is recognized as one of the most investigated lncRNAs [36]. MALAT-1 was initially identified in patients with non-small cell lung cancer (NSCLC), and subsequent investigations revealed its upregulation in tumors displaying a pronounced metastatic potential [37]. The primary sequence of the MALAT-1 gene spans over 8000 nucleotides (nt) and demonstrates a remarkable degree of conservation across multiple species [38]. In the human genome, the MALAT-1 gene is situated on chromosome 11q13, whereas in mice, it is localized to chromosome 19qA [39]. Furthermore, the expression level of MALAT-1 is notably elevated, comparable to several protein-coding genes, such as GADPH [36]. Similar to mRNA, MALAT1 is synthesized by RNA polymerase II (Pol II). The RNA precursor of MALAT-1 undergoes cleavage by RNase P, occurring promptly downstream of the poly(A)-rich tract. This cleavage event generates both the 3’ end of MALAT-1 and the 5’ end of a tRNA-like small RNA, known as MALAT-1-associated small cytoplasmic RNA (mascRNA) [40]. Following the cleavage of the 3’ end of mascRNA by RNase Z, a CCA sequence is added to the newly generated 3’ end [41]. The mature 3’ end of MALAT-1 displays a distinctive triple helix structure that distinguishes it from the conventional poly(A) tail. This unique structure arises from the presence of a genomically encoded A-rich tract along with two U-rich motifs [42]. MALAT-1 possesses remarkable stability and exhibits resistance to enzymatic cleavage due to the presence of its distinct triple helix structure at the 3’ terminus, which effectively shields the 3’ end from 3’-5’ exonuclease degradation [41]. Furthermore, the natural antisense transcript TALAM1 plays a role in enhancing the stability of MALAT-1 through the establishment of a feedforward positive regulatory loop [43]. MALAT-1 exhibits nuclear speckle accumulation, also known as interchromatin granule cluster, whereas mascRNA primarily undergoes translocation to the cytoplasm [44]. The mechanisms underlying the regulation of MALAT-1 turnover and the specific enzymes responsible for its degradation remain largely unclear. However, recent findings have revealed that the Drosha-DGCR8 complex, a crucial component involved in miRNA biogenesis, interacts with the 5’ end of MALAT-1, suggesting its involvement in modulating the abundance of MALAT-1 [45]. The subcellular localization of MALAT-1 implies its involvement in both physiological and pathological processes [46]. However, despite this, knockdown of MALAT-1 in mice does not induce discernible phenotypic alterations [47], potentially due to the lack of obvious effects exerted by MALAT-1 under normal conditions [48].

## MALAT-1 and cancer cells

**Fig. 1:**
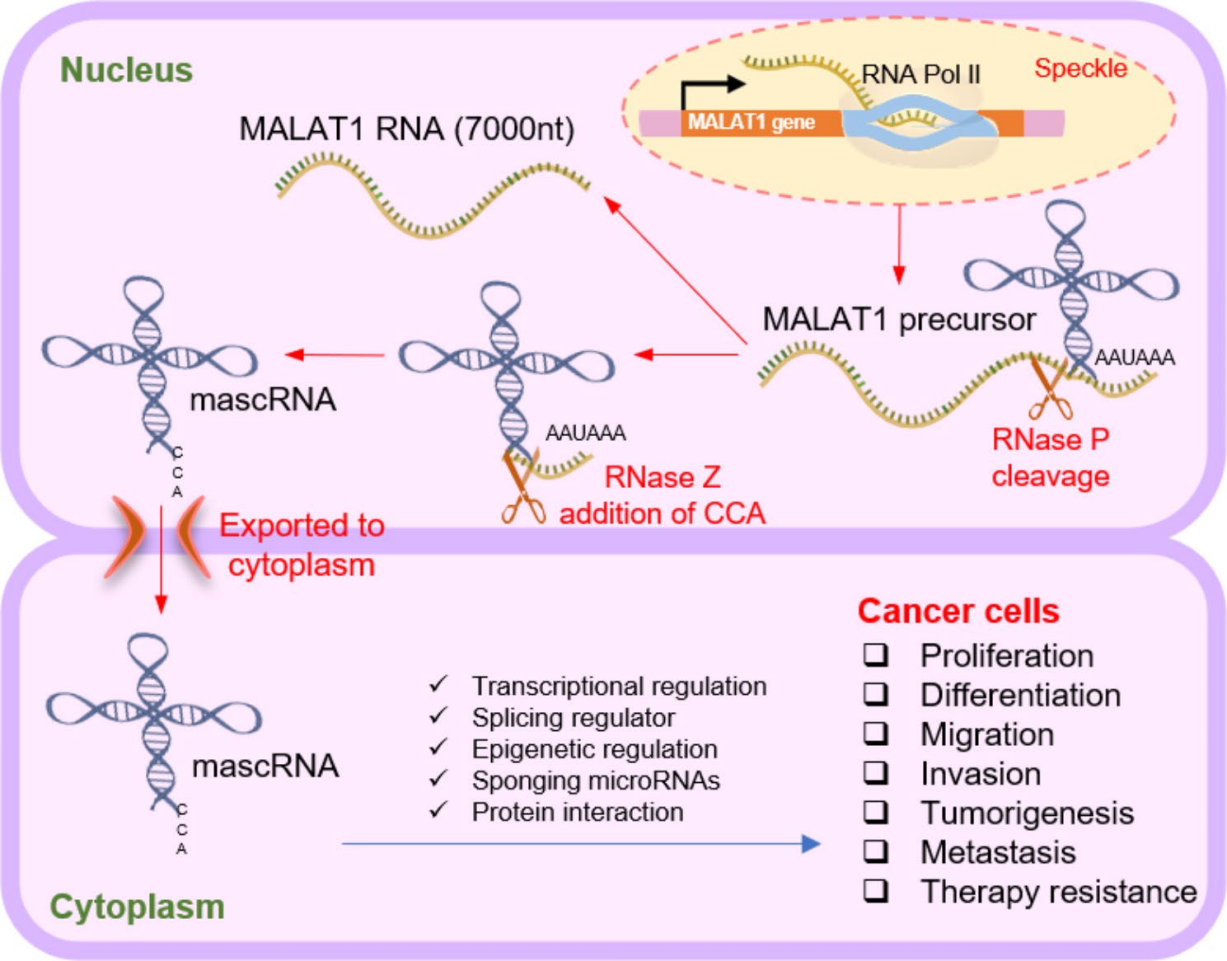
The intricate biogenesis process of MALAT-1 and provides insights into its diverse functions in cancer cells. MALAT1, a long non-coding RNA, is transcribed by RNA polymerase II (Pol II). The RNA precursor of MALAT-1 undergoes specific cleavage mediated by RNase P, which occurs immediately downstream of the poly(A)-rich tract. This cleavage event results in the generation of the 3’ end of MALAT-1 and the 5’ end of a small RNA molecule resembling transfer RNA, known as MALAT-1-associated small cytoplasmic RNA (mascRNA). Subsequently, the 3’ end of mascRNA undergoes further cleavage by RNase Z, followed by the addition of a CCA sequence to the newly generated 3’ end. The mature 3’ end of MALAT-1 exhibits a distinct triple helix structure that sets it apart from the conventional poly(A) tail. This unique structure is attributed to the presence of an A-rich region encoded in the genome, along with two U-rich motifs. The triple helix structure contributes to the stability and functional properties of MALAT-1. MALAT-1 has been implicated in various biological processes and has garnered significant attention as a key player in the development and progression of cancer. Its involvement in gene regulation, cellular signaling, and nuclear organization highlights its multifaceted role in cellular physiology and disease pathogenesis

MALAT-1 has been implicated in various biological processes and has emerged as a key player in cancer development and progression [49]. MALAT-1 is an important regulator of cell metabolism [50, 51] and in particular lipid metabolism [52]. MALAT-1 promotes angiogenesis and modulates immune cell infiltration [53, 54]. MALAT-1 has been shown to promote tumor growth by enhancing cell proliferation and inhibiting apoptosis [55]. It also contributes to tumor progression by facilitating angiogenesis, EMT, invasion, and metastasis [56]. It promotes metastasis by facilitating the migration, invasion, and colonization of cancer cells in secondary sites [57]. MALAT-1 influences the tumor microenvironment by modulating the communication between cancer cells and surrounding stromal cells [58]. It promotes the secretion of pro-inflammatory cytokines, growth factors, and extracellular matrix remodeling enzymes, which contribute to tumor progression and immune evasion [59]. Besides, MALAT-1 has been implicated in epigenetic regulation through its association with chromatin-modifying complexes. It can interact with proteins involved in DNA methylation, histone modification, and chromatin remodeling, leading to alterations in gene expression patterns [60]. MALAT-1 has been associated with resistance to chemotherapy and targeted therapies in various cancer types [61]. It can modulate the expression of drug resistance-related genes and contribute to the survival of cancer cells under therapeutic stress [62]. Elevated MALAT-1 expression has been correlated with poor prognosis in several cancer types [63]. Its overexpression has been linked to advanced disease stage, lymph node metastasis, and reduced overall survival rates [64]. Further research is needed to fully elucidate the molecular mechanisms by which MALAT-1 exerts its functional roles in cancer cells. Figure 1 provides a comprehensive depiction of the intricate biogenesis process of MALAT-1, highlighting its diverse functions within cancer cells and offering valuable insights into its role in oncogenesis.

## Functional significance of MALAT-1 in renal cell carcinoma

**Fig. 2:**
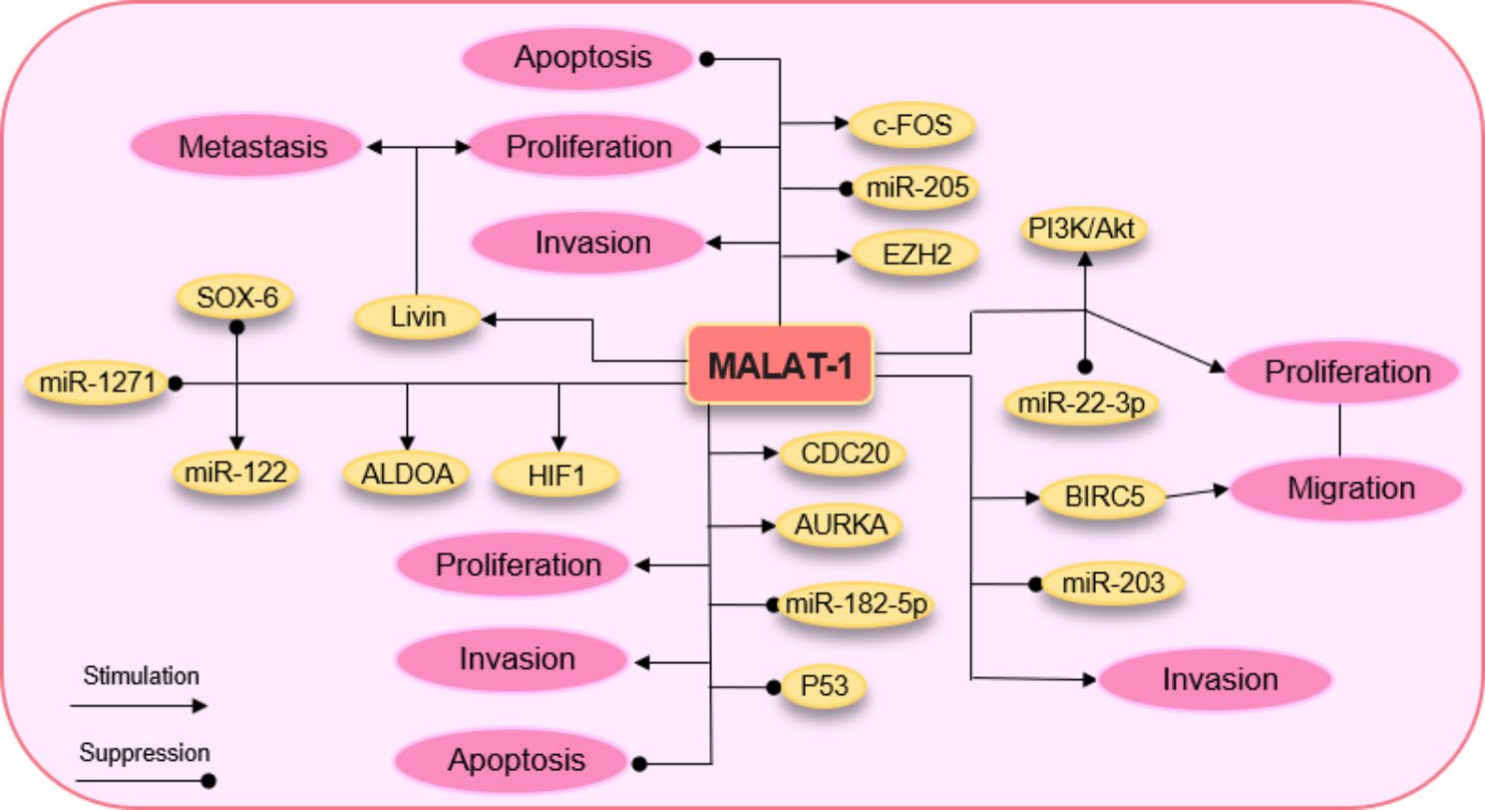
The intricate interplay between MALAT-1 and its downstream targets in renal cell carcinoma (RCC). The aberrant expression of MALAT-1 in RCC has been identified and linked to the advancement of tumors, invasion of surrounding tissues, and the formation of metastases. By functioning as a miRNA sponge and modulating the activity of transcription factors, MALAT-1 disrupts the normal gene expression profiles in RCC cells, thereby fostering the initiation and progression of the disease

The intricate regulatory network orchestrated by MALAT-1, encompassing its interactions with multiple miRNAs and transcription factors, has been reported to exert profound influence over the facilitation or inhibition of tumorigenesis in RCC (Table 1). Comparative analysis of RCC samples against normal renal tissue and cell lines revealed a notable up-regulation of MALAT-1. Employing a knockdown strategy to attenuate MALAT-1 expression yielded significant inhibition of cell proliferation and migration. Furthermore, the heightened expression of MALAT-1 has emerged as a potential prognostic indicator for unfavorable survival outcomes among RCC patients [65]. Zhang et al. for the first time evaluated the involvement of MALAT-1 in the cellular processes of ccRCC and its presence in ccRCC tissues. Their findings revealed significantly elevated levels of MALAT-1 expression in both ccRCC tissues and RC cells when compared to adjacent non-tumor tissues and normal human proximal tubule epithelial cells HK-2. By utilizing small interfering RNA (siRNA) to downregulate MALAT-1 expression, they observed a notable reduction in RCC proliferation, migration, and progression. The inhibition of MALAT-1 resulted in the acceleration of apoptosis, induced cell cycle arrest specifically in the G0/G1 phase, and reduced number of cells in S and G2/M phase. Knockdown of MALAT-1 expression demonstrated a significant inhibitory effect on the migration and invasion capabilities of RCC. Importantly, the researchers established a correlation between high MALAT-1 expression in ccRCC patients and advanced clinical features, as well as a shorter overall survival time compared to individuals with lower MALAT-1 expression levels. The upregulation of MALAT-1 exhibited significant correlations with tumor size, tumor stage, and lymph node metastasis, while no significant correlations were observed between this lncRNAs with patient’s age, gender, histological grade, or distant metastasis. This study demonstrated that the status of MALAT-1 expression independently predicted overall survival in ccRCC, suggesting that MALAT-1 could serve as a potential therapeutic target for inhibiting ccRCC progression [66]. Hirata et al. provided evidence showing elevated expression of MALAT-1 in human RCC tissues, and this upregulation was correlated with decreased overall survival rates among patients with RCC. Their results showed that MALAT-1 by targeting construct transcription factor gene (c-Fos) and Ezh2, and sponging miR-205 can induce RCC cell proliferation and invasion, and suppress apoptosis. miR-205 exhibits tumor-suppressive properties in renal cancer, acting as a crucial regulator, while its dysregulation has been documented across multiple human cancer types [67]. Significant downregulation of miR-205-5p expression was evident in comparison to the adjacent normal tissues, and this reduced expression demonstrated a pronounced association with adverse clinical outcomes. Notably, in vitro experiments substantiated that the overexpression of miR-205-5p led to diminished proliferation, invasion, and migration abilities of RCC. Moreover, the upregulation of miR-205-5p facilitated apoptosis induction and impeded the progression of EMT in RCC, a process that enables cells to acquire a more invasive phenotype and facilitates metastasis to distant sites. c-Fos serves as a pivotal transcription factor in the activation of MALAT-1. Elevated expression of Ezh2 and c-Fos was found to be significantly correlated with decreased overall survival among patients with RCC. Ezh2 functions as an enhancer of H3K27 methylation. Notably, following the knockdown of MALAT-1, there was a significant decrease observed in the levels of H3K27-me3, indicating the potential regulatory role of MALAT-1 in modulating H3K27 methylation. In RC tissues, a reciprocal association was detected between the protein expression of E-cadherin and the mRNA expression of MALAT-1. The use of si-MALAT-1 resulted in an elevation of E-cadherin expression, a reduction in β-catenin expression mediated by Ezh2, and subsequently led to decreased proliferation and invasion of RCC cells. A bidirectional interaction was identified between MALAT-1 and miR-205. This reciprocal relationship underscores the oncogenic role of MALAT-1 in RCC, highlighting its potential as a novel marker with significant implications in the understanding and characterization of this disease [68]. Chen et al. identified the biological roles and underlying mechanisms of MALAT-1 in RCC proliferation and metastasis. Their findings revealed elevated expression levels of MALAT-1 and the Livin protein in both RCC tissues and the RCC cell lines 786-O and Caki-1. Livin, also identified as ML-IAP (melanoma inhibitor of apoptosis protein), represents a constituent of the inhibitor of apoptosis protein (IAP) family that was originally associated with malignant melanoma [69]. The IAP family comprises a group of structurally related proteins that play significant roles in cancer development and therapeutic resistance [70]. It was discovered that MALAT-1 exerts a notable influence on the upregulation of Livin expression, leading to the initiation of cellular apoptosis and a subsequent decrease in cell viability. In order to delve deeper into the intricate functions of the MALAT-1 interaction, researchers conducted experiments wherein interference of MALAT-1 resulted in a reduction of cell viability and an augmentation of cell apoptosis in both RCC cell lines. The study team further validated their findings by successfully reversing the effects of si-MALAT-1 through the overexpression of Livin. Moreover, a noteworthy reduction in the size of si-MALAT-1-786-O cell xenografts was observed, indicating a significant suppression of tumor growth. Taken together, these findings suggest that the promotion of RCC proliferation and metastasis by MALAT-1 can be attributed to the upregulation of Livin expression [71]. Kulkarni et al. assessed the functional significance, mechanisms of action, and clinical relevance of miR-182-5p and MALAT-1 in the regulation of ccRCC. miR-182-5p exhibits a tumor suppressive function by modulating the expression of key protein regulators involved in the precise control of mitotic progression in ccRCC [72]. Their findings revealed that compared with adjacent normal tissues, miR-182-5p is downregulated in ccRCC due to CpG hypermethylation and decreased expression of key methylation regulatory genes such as DNMTl, DNMT3a, and DNMT3b. Therefore, miR-182-5p as a proapoptotic factor suppresses the tumorigenesis of ccRCC. In an RCC xenograft model, the intratumoral administration of miR-182-5p resulted in a significant reduction in tumor volume. it was discovered that MALAT-1, serving as a direct target of miR-182-5p, exhibited elevated expression levels in RCC. The study proposed that the regulatory impact of miR-182-5p on the proliferation of ccRCC could be ascribed to its capacity to repress the expression of the MALAT-1 gene. Through direct targeting of MALAT-1, overexpression of miR-182-5p resulted in the inhibition of cell proliferation, clonogenicity, and promotion of apoptosis (early apoptotic + late apoptotic), accompanied by G2/M phase cell cycle arrest. The overexpression of miR-182-5p led to a significant decrease in the expression levels of CDC20, AURKA, SKP2, and BMI1 as cell-cycle-related genes. Notably, downregulation of MALAT-1 led to increased expression of p53 (a downstream effector of miR-182-5p/MALAT-1) and decreased expression of CDC20 and AURKA, which are crucial players in the cell cycle mitotic phase. P53 has been documented to mediate cell-cycle arrest, cellular senescence, apoptosis, and transcriptional activation of miRNAs. The transient knockdown of MALAT-1 successfully replicated the effects observed with miR-182-5p overexpression. These findings underscore the potential roles of miR-182-5p and MALAT-1 in the regulation of ccRCC and provide valuable insights into their clinical significance [72]. Zhang et al. assessed the impact of MALAT-1 on the progression of RCC and observed a correlation between high MALAT-1 expression and elevated levels of BIRC5 in both RCC tissues and cell lines. BIRC5, also known as survivin, acts as an anti-apoptotic protein that hinders apoptosis-related signaling pathways, thereby promoting cell proliferation and facilitating cancer progression [73]. The researchers noted increased mRNA and protein expression of BIRC5 in RCC tissues. Additionally, in patients with kidney renal clear cell carcinoma (KIRC), higher BIRC5 levels were associated with poorer prognosis. Moreover, elevated expression of BIRC5 in RCC cell lines correlated with enhanced cell proliferation, invasion, and migration, as well as a decrease in the percentage of RCC cells in the G0/G1 phase. The study further revealed reduced expression of miR-203 in both RCC tissues and cell lines. By binding to BIRC5, miR-203 effectively decreased its expression, whereas the miR-203 inhibitor accelerated BIRC5 expression. Furthermore, MALAT-1 acted as a sponge for miR-203, influencing the expression of BIRC5, and ultimately promoting cell proliferation while reducing the percentage of RCC cells in the G0/G1 phase. In an in vivo study, high expression of MALAT-1 induced tumor growth, whereas downregulation of MALAT-1 suppressed tumor formation [34]. Li et al. showed that the expression of MALAT-1 in RCC tumor tissues and cell lines was higher than the control group. The findings indicated that MALAT-1 induced the activation of the PI3K/Akt signaling pathway and suppressed miR-22-3p, consequently promoting both the proliferation and migration of RCC. Further experiments revealed that short hairpin RNA (shRNA)‑mediated knockdown of MALAT-1 suppressed tumor cell viability and migration. The utilization of shRNA led to a substantial suppression of matrix metalloproteinase-3 (MMP-3) expression, concomitantly boosting the expression of migration and invasion inhibitory protein (MIIP), consequently impeding the proliferation and migration of RCC cells. Co-transfection of a miR-22-3p inhibitor partially counteracted the inhibitory effect of MALAT-1 shRNA on the expression of MALAT-1, revealing that the miR-22-3p inhibitor reciprocally elevated the level of MALAT-1. Besides, the introduction of MALAT-1 shRNA resulted in a significant inhibition of p-PI3K and p-Akt expression in RCC cells. However, the inclusion of a miR-22-3p inhibitor in the MALAT-1 shRNA-treated tumor cells significantly reversed the inhibitory effect of MALAT-1 shRNA on the expression of p-PI3K and p-Akt. Hence, the inhibition of MALAT-1 by shRNA resulted in the suppression of the PI3K/Akt signaling pathway activation, which was attributed to the upregulation of miR-22-3p expression. In vivo experiments demonstrated that the administration of MALAT-1shRNA effectively impeded the growth of RCC tumors, concurrently leading to an elevation in the expression of miR-22-3p [33]. Tumor-derived extracellular vesicles (EVs) have been implicated in fostering a pro-metastatic microenvironment. In a recent investigation by Jin et al., the role and mechanism of RCC cell 786-O-derived EVs (786-O-EVs) in RCC were examined. Initially, 786-O-EVs were isolated, characterized (CD63 + and CD81+), and observed to be internalized by RCC cells. The expression patterns of MALAT-1 were assessed before and after 786-O-EV treatment to evaluate RCC cell behavior. It was observed that 786-O-EVs enhanced malignant behaviors of RCC cells, concomitant with elevated levels of MALAT-1 expression. Notably, when MALAT-1 was knocked down in 786-O-EVs, the promotional effect of the EVs on RCC cells was reversed. Further investigation revealed that MALAT-1 exerted negative regulation on the transcription factor CP2 like 1 (TFCP2L1) by targeting ETS1 (ETS proto‑oncogene 1). TFCP2L1 exerts a pivotal influence on normal kidney development, actively modulating the intricate processes and molecular events that underlie the formation and maturation of the kidney throughout embryonic development [74]. ETS1, a member of the ETS (E26 transformation-specific) transcription factor family, occupies a crucial position in governing gene expression, encompassing a wide range of physiological and pathological processes such as development, cell proliferation, differentiation, apoptosis, and the intricate landscape of cancer progression [75]. Remarkably, in the context of cancer, ETS1 has emerged as a pivotal player, fostering tumor advancement and metastasis through its regulation of genes pivotal to cell invasion, angiogenesis, and the intricate orchestration of EMT [76]. 786-O-EVs facilitated the binding of ETS1 to the TFCP2L1 promoter, leading to decreased TFCP2L1 expression. In a lung metastasis model, 786-O-EVs promoted tumor growth and RCC lung metastasis, which could be mitigated by inhibiting MALAT-1. Hence, 786-O-EVs facilitated RCC invasion and metastasis by transporting MALAT1, thereby promoting the binding of the transcription factor ETS1 to the TFCP2L1 promoter [77]. Recent investigations have revealed that hypoxia inducible factor 1 (HIF1), a pivotal molecule involved in the pathogenesis of ccRCC [78], possesses the ability to target numerous metabolic enzymes and ncRNAs [79]. ALDOA, also known as fructose-bisphosphate aldolase A, a crucial target of HIF1, plays a pivotal role as a highly prevalent glycolytic enzyme [80]. Elevated expression levels of ALDOA have been significantly associated with unfavorable prognosis and reduced overall survival rates in patients diagnosed with ccRCC [81]. The researchers assessed the expression levels of ALDOA, SOX-6, mir-122, mir-1271, and MALAT-1. Remarkably, the up-regulation of HIF1 coincided with elevated expression levels of ALDOA, MALAT-1, and mir-122. Conversely, a decrease in the expression of mir-1271 and SOX-6 was observed. The functional significance of mir-1271 in the context of EMT and tumor invasion is paramount, as it exerts essential roles in suppressing these processes [82]. It has been shown that MALAT-1 by targeting mir-1271 as a molecular sponge can suppress inhibit its function to facilitate the tumorigenesis and invasion of tumor cells [83]. The acceleration of tumorigenesis has been reported to be facilitated by mir-122, which acts through the PI3K/Akt pathway and targets occludin as a means of exerting its regulatory effects [84]. SOX-6, known for its tumor suppressive properties across multiple malignancies, exhibits the ability to inhibit tumor development [85]. It has been discovered that both mir-122 and mir-1271 can effectively target and regulate the expression of SOX-6. Therefore, targeting of ALDOA, mir-122, and MALAT-1 suppression could hold potential therapeutic value for specific individuals affected by ccRCC [86]. Figure 2 illustrates the interplay between MALAT-1 and its downstream targets in RCC, providing valuable insights into the molecular mechanisms underlying MALAT-1-mediated effects in RCC.

**Table 1:** Signaling Pathways Associated with MALAT-1 in Renal Cell Carcinoma

MALAT-1			Results	Ref.
Tissue sample/cell line/animal model	Stimulation	Suppression		
Human proximal tubule epithelial cells HK-2, 106 primary ccRCC tissues from nephrectomy, Human RCC lines 786- O, ACHN, Caki-1, Caki-2	…	…	High MALAT-1 expression in both ccRCC tissues and RCC. MALAT-1 promoted RCC proliferation, migration, invasion, and progression. The inhibition of MALAT-1 accelerated apoptosis and induced cell cycle arrest in the G0/G1 phase. Shorter overall survival time in ccRCC patients.	[66]
50 patients (34 male and 16 female) with clear cell RCC, Normal renal epithelial cells (HK-2), human RCC lines 786-O, A-498, Caki-1, Caki-2	Ezh2, c-Fos	miR-205	MALAT-1 by targeting c-Fos and Ezh2, and sponging miR-205 can induce RCC cell proliferation and invasion, and suppress apoptosis.	[68]
Human tissues, RCC 786-O and Caki-1 cell lines, Six female athymic BALB/c nude mice	Livin	…	High MALAT-1 and the Livin protein in both RCC tissues and the RCC cell lines. MALAT-1 induced proliferation and metastasis.	[71]
Human RCC cell lines ACHN, 786-O, Caki-1, ccRCC nude mouse	CDC20, AURKA	miR-182-5p, P53	miR-182-5p reduced MALAT-1 expression. Overexpression of miR-182-5p by targeting MALAT-1 reduced tumor growth.	[72]
786-O, Caki-1, Ketr-3, RT112, T24, Ect1/E6E7	PI3K/Akt	miR‑22‑3p	MALAT-1 induced the activation of the PI3K/Akt signaling pathway and suppressed miR-22-3p to promote the proliferation and migration of RCC	[33]
HK-2, A498, 786-O, OS-RC-2, CAKI-1, HK-2, HEK293T, 786-O, BALB/c nude mice	BIRC5 (survivin)	miR-203	MALAT-1 by suppressing miR-203, influencing the expression of BIRC5, and promoted cell proliferation, invasion, and migration.	[34]
786-O, A498, ACHN, BALB/C nude mice	ETS1	TFCP2L1	786-O-EVs, as carriers of MALAT1, played a pivotal role in promoting RCC invasion and metastasis, facilitating the binding of the transcription factor ETS1 to the TFCP2L1 promoter.	[77]
Tumor and adjacent normal tissue samples from 14 patients with ccRCC	HIF1, ALDOA, miR-122	miR-1271, SOX-6	High expression of HIF1 was associated with elevated expression of ALDOA, MALAT-1, mir-122, and low expression of mir-1271 and SOX-6.	[86]

RCC: renal carcinoma cells, MALAT-1: Metastasis-associated lung adenocarcinoma transcript‐1; RIP: RNA binding protein immunoprecipitation; ccRCC: clear cell renal cell carcinoma. TFCP2L1: transcription factor CP2 like 1; ETS1: ETS proto‑oncogene 1;

## Future perspective

**Fig. 3:**
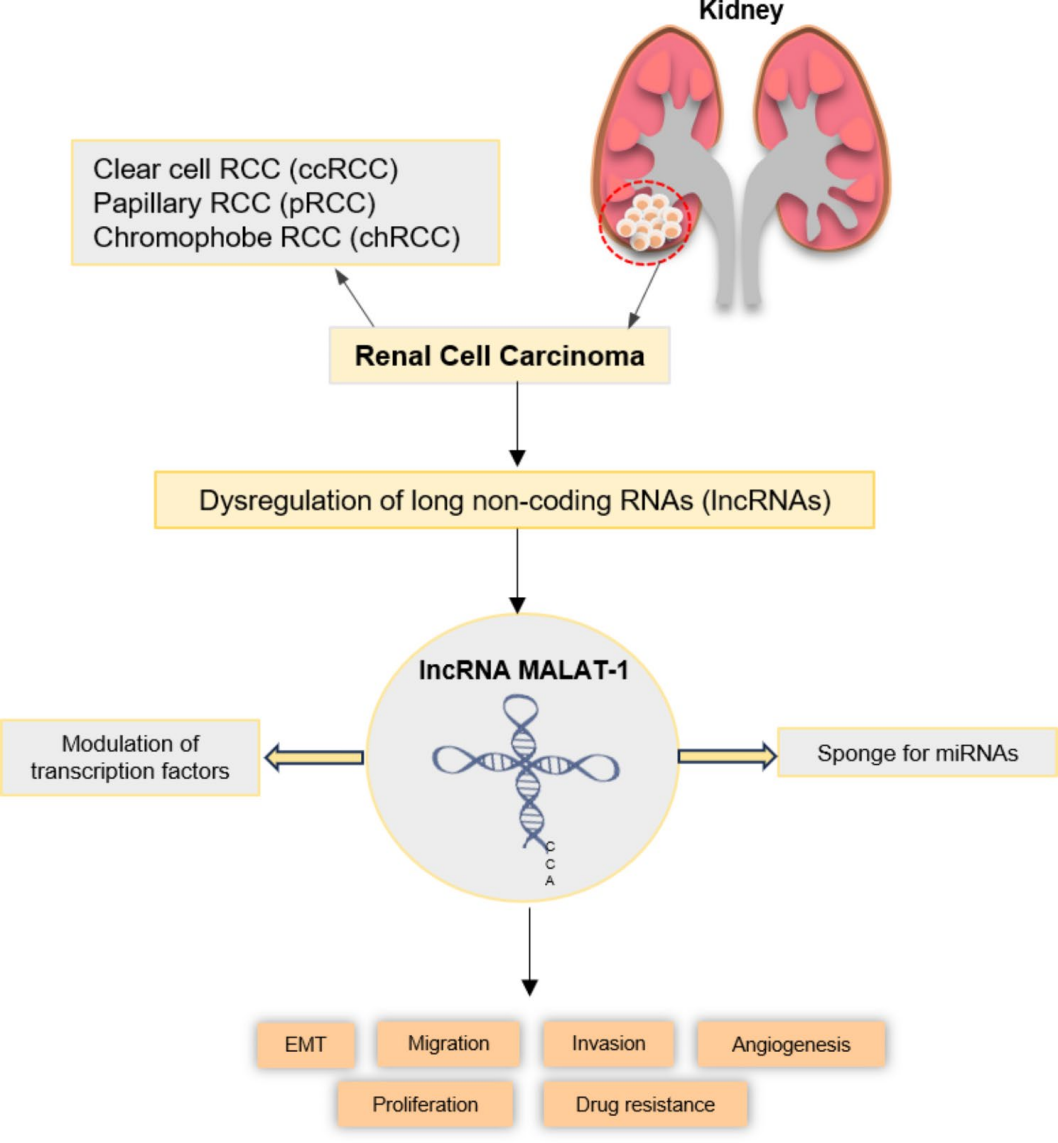
MALAT-1 plays important roles in RCC development and progression

MALAT-1 has emerged as a crucial player in various facets of RCC advancement and metastasis (Fig. 3). Its involvement encompasses tumor growth, invasion, angiogenesis, drug resistance, and unfavorable clinical prognosis. Through targeted interactions with specific proteins and signaling pathways implicated in cell cycle regulation, MALAT-1 has been observed to stimulate cell proliferation and tumor growth in RCC, thereby fostering enhanced cell division and overall tumor progression. Additionally, MALAT-1 has the ability to induce EMT and facilitate the metastatic dissemination of RCC cells to distant anatomical sites. In the context of RCC, heightened angiogenesis plays a critical role in facilitating tumor growth and metastasis. Notably, MALAT-1 exhibits the ability to interact with factors intricately associated with the process of angiogenesis, thereby actively contributing to the establishment and maintenance of a pro-angiogenic microenvironment that supports RCC progression. Accumulating evidence indicates the potential involvement of MALAT-1 in imparting resistance to specific therapies employed in RCC. This lncRNA has been shown to exert regulatory control over the expression of genes implicated in drug metabolism and efflux, ultimately resulting in diminished drug sensitivity and the emergence of treatment resistance. Furthermore, the expression levels of MALAT-1 have been correlated with clinical outcomes in individuals diagnosed with RCC. High MALAT-1 expression has been linked to poor prognosis, including shorter overall survival and increased risk of recurrence and metastasis. It’s important to note that while these findings suggest the involvement of MALAT-1 in RCC, further research is needed to fully understand its precise mechanisms of action and its potential as a therapeutic target or diagnostic marker. Ongoing studies may provide additional insights into the functional roles of MALAT-1 in RCC and its clinical implications.

## Conclusion

In the current study, we have provided an overview of the functional implications of MALAT-1 in RCC. It has been established that MALAT-1 exerts its influence on RCC development and advancement by acting as a sponge for various miRNAs, including miR-205, miR-182-5p, miR-203, miR‑22‑3p, and miR-1271. Additionally, it is essential to consider the involvement of several transcription factors, such as Ezh2, c-Fos, Livin, CDC20, survivin, PI3K/Akt, and AURKA, as downstream target genes of MALAT-1 in the context of targeted therapy for RCC.

## Data Availability

The datasets used and/or analyzed during the current study are available from the corresponding author on reasonable request.
